# Editorial: Deafness, aging and Alzheimer's disease: Neurobiological links and therapy options

**DOI:** 10.3389/fnins.2022.1114383

**Published:** 2023-01-04

**Authors:** José M. Juiz, Verónica Fuentes Santamaría, Verena Scheper, Thomas Lenarz

**Affiliations:** ^1^Department of Otorhinolaryngology, Head and Neck Surgery, Hannover Medical School, Hanover, Germany; ^2^Cluster of Excellence “Hearing4all” of the German Research Foundation, DFG, Hanover, Germany; ^3^Instituto de Investigación en Discapacidades Neurológicas (IDINE)/School of Medicine, Universidad de Castilla-La Mancha (UCLM), Albacete, Spain

**Keywords:** cognitive impairment, hearing loss, presbycusis, cochlear implant, neurodegeneration

For decades, age-related hearing loss (ARHL) was intuitively considered one of many factors worsening Alzheimer's disease (AD). Intuition yielded way to epidemiological evidence, after an observational case/control study reported a strong association between ARHL and AD (Uhlmann et al., 1989). However, almost 30 years passed until the recent realization that ARHL is the main preventable risk factor of AD (Livingston et al., 2017; Loughrey et al., 2018). By removing ARHL there would be a 9.3% reduction of AD cases (Livingston et al., 2017). Somehow, this figure evokes causality for ARHL in a large absolute number of AD subjects. Despite this evidence, ARHL still appears “buried” among other risk factors of AD and its unique relevance continues to be overlooked in practice (Lin and Albert, 2014). This is mainly due to the lack of a body of evidence supporting neurobiological mechanistic links between ARHL and AD, mirroring epidemiological evidence (Griffiths et al., 2020; Johnson et al., 2021). Contributions to this Special Topic focus on this issue, covering research in humans and animal models relevant to understand interactions between ARHL and AD.

Recapitulating theories of sensory processing and perception in brain networks allows building testable hypothesis on interactions between ARHL and AD. Pérez-González et al. suggest that auditory deviance detection, by which unexpected sounds relevant for adaptive behavior are segregated from repetitive sounds, may link ARHL and AD. Deviance detection is degraded in AD patients, with difficulties perceiving auditory objects that change with time. Cholinergic neuromodulation is essential for deviance detection, and loss of cholinergic circuits is a signature of AD. Integrating network processing mechanisms with anatomical and neurochemical substrates holds promise in the search for mechanistic interactions between ARHL and AD.

Anatomical substrates linking ARHL, and AD are unknown. Llano et al. examined relationships between reported hearing loss and regional brain volumes in MRIs from non-cognitively impaired subjects and subjects with mild cognitive impairment or AD from the Alzheimer's Disease Neuroimaging Initiative database. Self-reported hearing loss correlates with lower volume and accelerated volume loss in the brainstem and cerebellum of subjects with AD. Therefore, AD pathology in the hindbrain may be triggered or potentiated by hearing loss, and this may amplify cognitive pathology. This highlight focusing on mechanistic links between ARHL and AD beyond the forebrain and neocortex.

Beyond anatomical substrates, Johne et al. report altered neuronal and network activities in the inferior colliculus and medial prefrontal cortex in rats deafened with neomycin. In the latter region, key for cognitive processing, single unit activity is reduced and irregular after hearing loss. Moreover, network activity shows reduced theta and enhanced gamma bands, both linked to cognitive disturbances. Interestingly, there are only minor behavioral deficits in deaf rats, suggesting that some aspects of cognitive function may be more affected than others by hearing loss or that ARHL involves additional mechanisms not reproduced by neomycin deafening. Unraveling functional neuronal circuits linking ARHL with cognitive impairment is an essential future avenue.

The search for basic cell and tissue pathological mechanisms linking ARHL and AD is emphasized by Alvarado et al. A pivotal role of oxidative stress in triggering and maintenance of ARHL and AD is one testable mechanism linking both, which will potentiate each other. Frailty syndrome is also linked to oxidative stress and worsens with ARHL, thus directly and indirectly amplifying the interactions between ARHL and AD. This calls for untangling complex biological interaction networks through robust experimental models and clinical studies.

A pathological signature of ARHL, at least in many animal models, is cochlear synaptopathy, i.e., uncoupling of afferent synapses from auditory receptor cells. Work by Savitska et al. supports that successful central compensation of temporal auditory processing deficits following cochlear synaptopathy, depends on an efficient corticosterone-mediated stress response. If auditory processing in the aging brain is affected by the quality of the stress response, this may be relevant in view of links between AD and stress.

The phantom auditory perception of tinnitus is often associated with ARHL. This association may further impact AD. Scott et al. show that behavioral manifestations of salicylate-induced tinnitus are reduced by treatment with BMS-191011, an agonist of the large conductance calcium-activated potassium channel, key regulator of neuronal network excitability. Results point to excitability modulation in the inferior colliculus as a mechanism of action. Advances in tinnitus research should be aligned with research in cognitive impairment and AD.

Biomarkers for traits of ARHL predicting cognitive impairment and AD are needed. Gommeren et al. searched systematically for publications on central auditory processing measured by cortical auditory evoked potentials (CAEPs) in subjects with mild cognitive impairment or AD, with and without hearing loss, showing at least one auditory event-related potential. No studies were found reporting both evaluation of hearing and cognitive status in CAEPs in subjects with mild cognitive impairment or AD. This “evidence gap” hinders advances in understanding interactions between ARHL and AD of immediate clinical relevance.

Research efforts converge on efficient treatments of ARHL to reverse or attenuate AD. The cochlear implant is the most successful neural prosthesis available. In ARHL it may impact AD. For this, it is essential to optimize outcomes of cochlear implantation in the elderly, as discussed by Illg and Lenarz. They authoritatively highlight factors crucial for such optimization. Management of residual low frequency hearing is a priority, which conditions electrode insertion and electroacoustic simulation. Comorbidities must be considered. Pre-existing cognitive decline or impairment call for timely implantation. Research will be aimed at identifying additional success predictors and learning curves in the elderly with or without cognitive impairment.

Contents highlight a promising, though long way ahead. Concerted actions require multi-level testable hypothesis (Figure 1) (Griffiths et al., 2020; Slade et al., 2020; Nadhimi and Llano, 2021), robust animal models reproducing ARHL and AD traits, strong translational interactions among the clinic, cochlear implant and hearing aid stakeholders and research labs, as well as focused systematic reviews and metanalysis, among others. Research, with involvement of patients and families, will result in significant improvements in life quality and disease burden.

**Figure 1:**
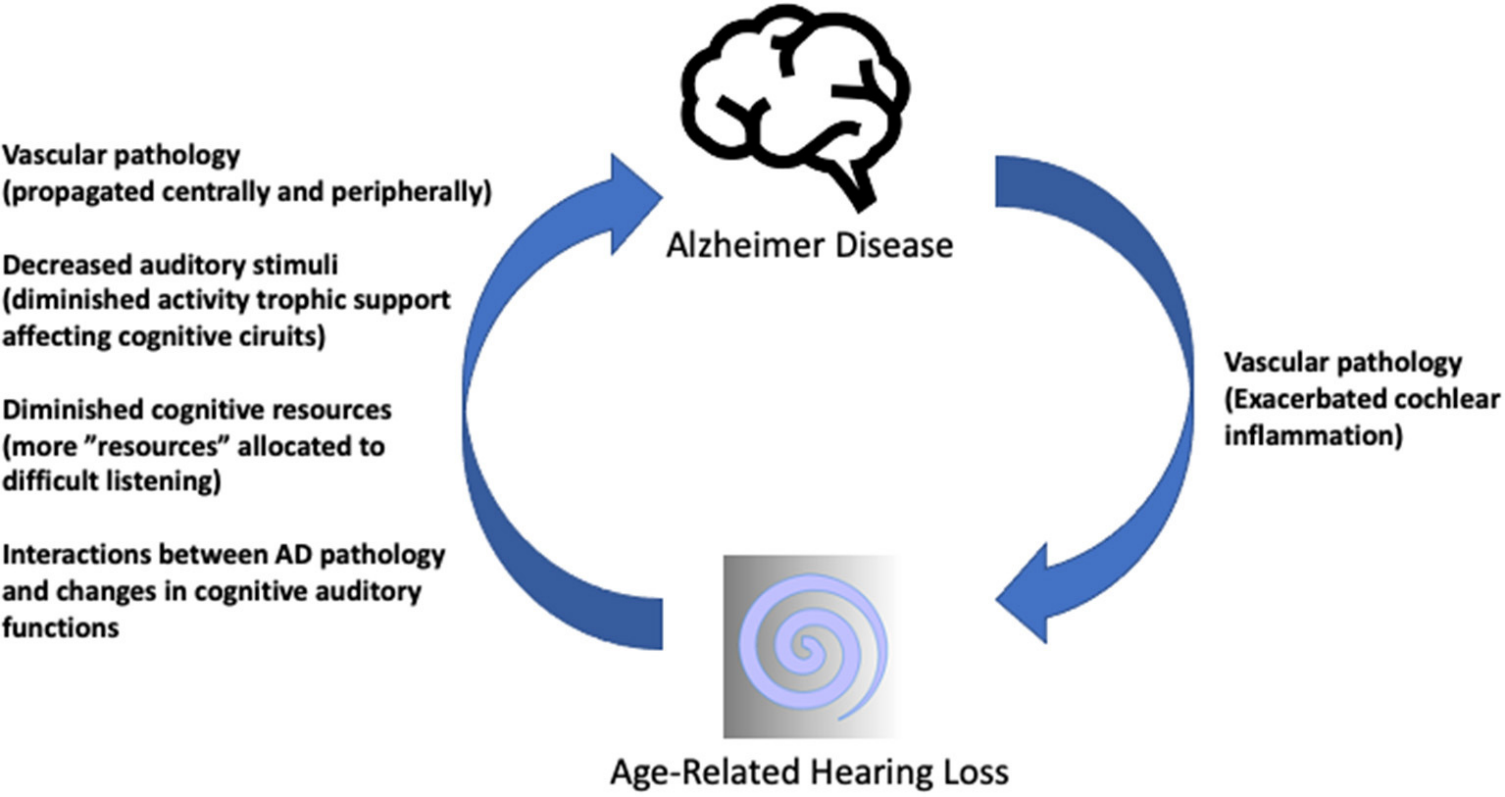
Summary sketch of possible interactions between ARHL and AD. Hypothetic mechanisms of ARHL influencing AD (ascending arrow) have been covered in detail (Griffiths et al., 2020; Nadhimi and Llano, 2021). The descending arrow represents less often considered influences of AD pathology on the auditory receptor which may close the loop of a self-sustaining pathophysiological vicious circle. AD-related or pre-existing vascular pathologies may propagate to the cochlear microvasculature, exacerbating mechanisms involved in age-related “inflammaging” in the cochlea, as preliminarily reported (Juiz et al., 2022) resulting in further decreased central auditory input. Schematic drawings under Creative Commons license CCBY 3.0 and CCBY-SA-3.0.

## Author contributions

JJ drafted the manuscript. VF, VS, and TL contributed input and review. All authors contributed to the article and approved the submitted version.
